# Clinical and biological relevance of glial fibrillary acidic protein in Alzheimer’s disease

**DOI:** 10.1186/s13195-023-01340-4

**Published:** 2023-11-03

**Authors:** Zhengshi Yang, Karthik Sreenivasan, Erin N. Toledano Strom, Amanda M. Leisgang Osse, Lorenzo Gabriel Pasia, Celica Glenn Cosme, Maya Rae N. Mugosa, Emma Léa Chevalier, Aaron Ritter, Justin B. Miller, Dietmar Cordes, Jeffrey L. Cummings, Jefferson W. Kinney

**Affiliations:** 1 Cleveland Clinic Lou Ruvo Center for Brain Health, Las Vegas, NV USA; 2 Department of Brain Health, University of Nevada Las Vegas, Las Vegas, NV USA; 3 Kirk Kerkorian School of Medicine, University of Nevada Las Vegas, Las Vegas, NV USA; 4 Hoag’s Pickup Family Neurosciences Institute, Newport Beach, CA USA; 5 Department of Psychology and Neuroscience, University of Colorado, Boulder, CO 80309 USA; 6 Chambers-Grundy Center for Transformative Neuroscience, Pam Quirk Brain Health and Biomarker Laboratory, Department of Brain Health, University of Nevada Las Vegas, Las Vegas, NV USA

**Keywords:** Glial fibrillary acidic protein, Alzheimer’s disease, Ptau-181, ATN framework, 18F-AV45

## Abstract

**Introduction:**

There is a tremendous need for identifying reliable blood-based biomarkers for Alzheimer’s disease (AD) that are tied to the biological ATN (amyloid, tau and neurodegeneration) framework as well as clinical assessment and progression.

**Methods:**

One hundred forty-four elderly participants underwent 18F-AV45 positron emission tomography (PET) scan, structural magnetic resonance imaging (MRI) scan, and blood sample collection. The composite standardized uptake value ratio (SUVR) was derived from 18F-AV45 PET to assess brain amyloid burden, and the hippocampal volume was determined from structural MRI scans. Plasma glial fibrillary acidic protein (GFAP), phosphorylated tau-181 (ptau-181), and neurofilament light (NfL) measured by single molecular array (SIMOA) technology were assessed with respect to ATN framework, genetic risk factor, age, clinical assessment, and future functional decline among the participants.

**Results:**

Among the three plasma markers, GFAP best discriminated participants stratified by clinical diagnosis and brain amyloid status. Age was strongly associated with NfL, followed by GFAP and ptau-181 at much weaker extent. Brain amyloid was strongly associated with plasma GFAP and ptau-181 and to a lesser extent with plasma NfL. Moderate association was observed between plasma markers. Hippocampal volume was weakly associated with all three markers. Elevated GFAP and ptau-181 were associated with worse cognition, and plasma GFAP was the most predictive of future functional decline. Combining GFAP and ptau-181 together was the best model to predict brain amyloid status across all participants (AUC = 0.86) or within cognitively impaired participants (AUC = 0.93); adding NfL as an additional predictor only had a marginal improvement.

**Conclusion:**

Our findings indicate that GFAP is of potential clinical utility in screening amyloid pathology and predicting future cognitive decline. GFAP, NfL, and ptau-181 were moderately associated with each other, with discrepant relevance to age, sex, and AD genetic risk, suggesting their relevant but differential roles for AD assessment. The combination of GFAP with ptau-181 provides an accurate model to predict brain amyloid status, with the superior performance of GFAP over ptau-181 when the prediction is limited to cognitively impaired participants.

**Supplementary Information:**

The online version contains supplementary material available at 10.1186/s13195-023-01340-4.

## Introduction

In the last two decades, biomarkers have been developed to support the diagnosis of Alzheimer’s disease (AD) in living patients, and the field is rapidly shifting from the syndromal definition toward the biological definition of AD [1]. Positron emission tomography (PET) and structural magnetic resonance imaging (MRI) are the well-recognized techniques to evaluate pathological hallmarks of AD, including amyloid (A), tau (T), and neurodegeneration (N) [2]. However, their availability and cost make them challenging for all populations and clinics to readily access.

PET and MRI outcomes are leveraged to evaluate the viability of cerebrospinal fluid (CSF) biomarkers as well as novel blood-based biomarkers [3–6]. CSF amyloid-beta protein 42 (Aβ42) concentration is highly consistent with the metric derived from brain amyloid PET imaging [3]. CSF phosphorylated tau (ptau) protein and tau PET imaging are strongly correlated [5], and CSF total tau protein is associated with brain atrophy and cognitive decline [7]. While there is great concordance of CSF markers with pathology related to clinical and imaging measures, the requirement of lumbar puncture for collecting CSF sample hinders its widespread use. Despite considerable progress in understanding AD pathological hallmarks, more efforts are still needed to identify cost-effective and easily-accessible biomarkers for screening, prognosis/staging, and treatment response. Blood-based biomarkers have emerged as candidates for this role, with less cost compared to PET and less-invasive compared to lumbar puncture.

Pathological hallmarks in the brain have been found to be associated with the surrogates in the blood. Plasma neurofilament light (NfL), as a potential marker of neurodegeneration, was associated with the future progression of cognitive decline, brain atrophy, and hypometabolism [8]. Plasma ptau-181 was correlated with tau-PET [9] and used to stratify the tau pathological changes [10]. Emerging data showed that plasma Aβ42/40 ratio was predictive of brain amyloid status [11]; however, the data on blood-based amyloid is still mixed [4, 12]. The lack of reliable blood-based biomarkers for AD is a substantial barrier to the development of clinical therapeutics.

Recently, more attention has been focused on plasma proteins from pathological pathways which interact with conventional AD pathologies. Activated astrocytes are part of the inflammatory process in AD [13]; they are observed to surround amyloid plaques in the AD brain [14], suggesting the close relationship between astrocytosis and amyloid pathology. The process of astrocytosis is characterized by the upregulation of the intermediate filament protein glial fibrillary acidic protein (GFAP). Plasma GFAP concentration has been correlated with cortical amyloid status derived from 18F-flutemetamol amyloid PET scans [15, 16], and the longitudinal change in GFAP was associated to the development of clinical AD and cognitive decline [17]. However, a comprehensive assessment of GFAP in a single cohort including its relevance to the complete ATN framework and clinical diagnosis/progression remains to be conducted. Determining the associations of GFAP with an existing tau marker and neurodegenerative metric, together with brain amyloid, could be helpful toward establishing a better understanding of the potential multi-faceted role of astrocytosis as well as determining if GFAP serves as a viable blood-based biomarker in AD. In addition, the differential roles of GFAP and other blood-based biomarkers need to be clarified.

This study aims to provide a comprehensive assessment of GFAP and its relevance to several pathological or clinical measures in AD, illustrate the relevant but differential roles of various blood-based proteins, and clarify the influence of demographical variables on their concentrations independent of disease progression. Plasma protein concentrations including GFAP, ptau-181, and NfL, together with amyloid PET and structural MRI scans, were acquired from the participants in the cohort. To achieve the goals, the group comparisons between participants stratified by brain amyloid status and cognitive impairment or apolipoprotein E (APOE) genotype were conducted; the association of proteins with ATN markers, cognition, and demographical variables including age, sex, and education were evaluated; and the capabilities of these proteins in predicting future functional decline or brain amyloid positivity were tested. We hypothesized that (1) GFAP has a multi-faceted role in characterizing AD pathological hallmarks, and (2) GFAP provides valuable knowledge about the disease complementary to other plasma biomarkers, instead of replaceable by other markers.

## Methods

### Subjects

The data used in this study were collected from the Nevada Center for Neurodegeneration and Translational Neuroscience (CNTN, https://nevadacntn.org/). The CNTN is a longitudinal, natural history study consisting of an annual clinical examination, neuropsychological assessment, and MRI/PET acquisition. The study was approved by Cleveland Clinic Institutional Review Board and all participants have given written, informed consent.

The baseline cognitive assessment includes widely accepted clinical instruments including the Clinical Dementia Rating (CDR), Alzheimer’s Disease Assessment Scale-cognitive subscale (ADAS-cog), Functional Assessment Staging (FAST) [18], Mini Mental State Examination (MMSE), and Montreal Cognitive Assessment (MoCA). An 18F-AV45 PET (AV45-PET) scan was conducted at baseline to determine brain amyloid status. Individuals with mild cognitive impairment (MCI) or mild dementia were recruited from the Memory Disorders clinic at the Cleveland Clinic Lou Ruvo Center for Brain in Las Vegas, Nevada, while the cognitively unimpaired cohort was recruited from local community resources. A total of 144 participants from the CNTN cohort having 18F-AV45 PET scan, structural MRI scan, and blood sample available were included in the analysis, comprising 57 cognitively unimpaired (CU) and 87 cognitively impaired (CI, including both MCI and mild dementia) individuals. Participants’ diagnoses were made based on a clinical committee who reviewed each participant’s CDR performance, neuropsychological testing, and clinical data. Diagnoses were rendered based on NIA-AA criteria [1]: participants were determined to be cognitively normal if global CDR score was 0 and had no neuropsychological tests 1.5 standard deviations below age and education norms, MCI if they had CDR scores of 0.5 or 1 and still functioned independently in the community, and mild dementia (CI) if they had CDR of 0.5 or 1 and neuropsychological test impairments in more than one cognitive domain and not able to live independently in community. The demographic characteristics of participants are summarized in Table 1. The differences between CU and CI group were examined by chi-squared test for categorical variables (e.g., sex, race, ethnicity and APOE) and by Kruskal–Wallis test for continuous variables (e.g., age, years of education and global CDR). Compared to CU participants, the CI individuals were slightly older (*p* = 0.02), had more APOE ε4 carriers (*p* = 0.004), and had higher CDR scores (*p* < 0.001). Sex, years of education, race, and ethnicity did not show difference between CU and CI groups.

**Table 1:** Subject characteristics at baseline

Variable	Cognitively unimpaired (CU) *n* = 57		Cognitively impaired (CI) *n* = 87		CU versus CI
	CU − *n* = 46	CU + *n* = 11	CI − *n* = 26	CI + *n* = 61	*p* value
**Age**	70.74 ± 6.54	72.00 ± 6.36	71.46 ± 6.83	74.21 ± 6.43	0.02
**Sex**	21 M/25F	7 M/4F	15 M/11F	35 M/26F	> 0.05
**Years of education**	16.35 ± 2.54	15.91 ± 2.47	15.92 ± 2.62	15.46 ± 2.71	> 0.05
**Race (White)**	40	11	24	58	> 0.05
**Ethnicity (non-Hispanic)**	44	8	26	57	> 0.05
**% of APOE ε4 carrier**	28%	45%	16%	78%	0.004
**Global CDR**	0.09 ± 0.22	0.14 ± 0.23	0.50 ± 0.26	0.58 ± 0.24	< 0.001

### APOE genotyping

Whole blood was collected at baseline into PAXgene Blood DNA Tubes (PreAnalytiX, Hombrechtikon, Switzerland). DNA was extracted from the whole-blood samples using the PAXgene Blood DNA Kit according to kit specifications. DNA concentration and purity were assessed using a NanoDrop spectrophotometer (Thermo Scientific, Wilmington, DE, USA). More detailed description of the APOE genotyping protocol was provided in our previous report [19]. Based on the APOE genotype, each individual was categorized as a non-ε4 carrier (no ε4 allele) or an ε4 carrier (one ε4 allele or two ε4 alleles).

### Plasma GFAP, NfL, and ptau-181 concentrations

Whole blood was collected in K2-EDTA tubes that were centrifuged at 2000 × g for 10 min at 4 °C in which the plasma was aliquoted for storage at – 80 °C. Biomarkers were measured from plasma samples on the Quanterix Single Molecular Array (SIMOA) HD-X platform. Calibrators were run neat and measured in triplicate (except for the GFAP kit, which were run in duplicate), while controls and samples were run via the 4 × instrument dilution method and measured in duplicate. The Quanterix Simoa GFAP Discovery Kit (cat# 102,336) was used quantify the mean concentration of GFAP in patient plasma samples. Advantage V2 Kit (cat# 103,714) was used to quantify the mean concentration of ptau-181. The Quanterix Simoa NF-light Advantage Kit (cat# 103186) was used to quantify the mean concentration of NfL in patient plasma samples.

### Structural MRI and amyloid PET

A high resolution (1 mm × 1 mm × 1 mm) structural image was acquired on a 3 T Siemens Skyra scanner using a T1-weighted gradient echo 3D MP-RAGE sequence (TR = 2300 ms, TE = 2.96 ms, flip angle = 9°). Amyloid PET data were acquired from all subjects after injection of 370 MBq (± 10%) of florbetapir (18F-AV45) for 20 min at 50–90 min after injection. A single Biograph mCT scanner (Siemens Healthcare, Malvern, PA, USA) was used for acquiring PET data.

All T1-weighted images were visually inspected for motion corruption and tissue contrasts. Each T1-weighted image was input to FreeSurfer 6.0 image analysis suite to perform cortical labeling and volumetric segmentation. Regional volumes (mm^3^) were extracted for the hippocampus and all the other regions identified in the Desikan-Killiany atlas [20]. To address the head size differences among participants, the relative volume, instead of the absolute volume, of the hippocampus was used in the analyses presented here. The relative volume of the hippocampus was computed as the total absolute volume of the left and right hippocampus normalized by the total intracranial volume with an arbitrary scaling factor of 1000.

T1-weighted images (acquired closest to the amyloid PET images) were used as a structural template to define the reference region in the native space for each subject. The PET scans for each subject were co-registered to their structural MRI scans, and subsequently, regional standardized uptake value ratios (SUVRs) were computed by averaging the signal from gray matter voxels in each region and then normalized to the average signal in the whole cerebellum. Following a previously published AV45-PET processing pipeline [21], the composite SUVR, an overall assessment of brain amyloid, was computed by averaging the SUVRs from frontal, anterior/posterior cingulate, lateral parietal, and lateral temporal regions. Amyloid positivity was determined by the composite SUVR with the cutoff of 1.11.

### Statistical analysis

The plasma concentrations of GFAP, NfL, and ptau-181 (pg/ml) were not normally distributed based on Anderson–Darling test (*p* < 0.001) and were log-transformed to better approximate the normal distribution before any statistical analyses were conducted (*p* > 0.15 after transformation). To assess the influence of clinical diagnosis (CU/CI), amyloid status, and demographical variables including age, sex, and education on blood-based markers, a linear regression model was used in the analysis with the concentration of plasma marker as the response variable and clinical diagnosis, amyloid positivity, age, sex, and education as predictor variables, namely plasma marker ~ clinical diagnosis + amyloid positivity + age + sex + education, with or without the interaction term between clinical diagnosis and amyloid positivity. Age, sex, amyloid positivity, and its interaction term with clinical diagnosis were found to be significantly associated with at least one plasma marker (see the “Results” section).

With the findings from the analysis above, we then conducted post hoc two-sample *t*-tests to examine the concentration differences between groups stratified by clinical diagnosis and amyloid positivity, namely amyloid negative CU (CU −), amyloid positive CU (CU +), amyloid negative CI (CI −), and amyloid positive CI (CI +) groups, after adjusting for demographical variables. Adjusted concentrations for these plasma markers were used in all following group comparisons and association analysis, except the association analysis with demographical variables since they are the variables of interest, where the original concentrations were used in the analysis. Bonferroni correction was used to control for multiple tests; corrected significance levels were reported in the “Results” section unless otherwise stated.

APOE ε4 allele is the strongest genetic risk factor in sporadic AD population [22]; APOE genotype and amyloid load play as the confounding factors of each other. In addition, the CI participants had higher proportion of ε4 carriers in the cohort. Because of these reasons, a separate group comparison was conducted with the two-sample *t*-test to examine if these plasma markers were differed by APOE genotype (non-ε4 versus ε4 carriers) across all participants or among participants stratified by clinical diagnosis or amyloid positivity.

Since sex and age were significantly associated with the concentrations of some plasma markers, we evaluated the differences between female and male separately in CU − , CU + , CI − , and CI + groups by two-sample *t*-tests. We then tested the age effect on these plasma markers and whether the age effect was altered by AD pathology. In detail, Pearson’s correlation (*r*) was calculated separately for CU − participants and the participants under AD pathology (namely Amy +) or across all participants. The Fisher’s *z* statistic was applied to test if the correlations were different between CU − and Amy + groups following Fisher’s r-to-z transformation, and the *r*^*2*^ value indicates the percentage of variance explained by age for these three markers. The original concentrations of these plasma markers were used in the analysis since age and sex were the factors of interest in the analysis.

One important aspect to test if a plasma marker is useful in AD is to test its relevance to the biological ATN framework. The composite SUVR from AV45-PET and the hippocampal volume derived from T1 structural MRI scan were well-recognized imaging biomarkers to characterize amyloid pathology and neurodegeneration, respectively. In the cohort, tau PET scan was not available; thus, the peripheral proximity of tau pathology, plasma ptau-181, was used in the analysis. Correlation analysis was conducted to examine how strongly these plasma markers were correlated to the ATN markers, namely AV45 composite SUVR, ptau-181, and hippocampal volume. Spearman’s correlation was used for AV45 composite SUVR due to its bimodal distribution. Pearson’s correlation was used for all the other measures. The correlation between ptau-181 with the T marker was omitted since ptau-181 was treated as the T marker.

The clinical relevance of these blood-based proteins was investigated from two different aspects by (1) illustrating their association with clinical measures and (2) assessing their prognostic power in predicting future functional decline in the follow-up visit based on Functional Assessment Staging (FAST) scale scores (ranging from 1 to 7) [18]. A correlation analysis between fluid biomarkers and ADAS-cog were carried out. Considering that the rate of cognitive decline in AD accelerates with disease progression [23], an individual with lower baseline FAST score is more likely to progress slower. To better evaluate how well plasma markers predict future functional decline, we selected a subsample of participants having the same baseline FAST score to avoid the bias induced by baseline functional status. Among the 144 participants, baseline FAST as 3 (mild functional losses; the stage of objective functional deficits interfering with a person’s most complex tasks) had the highest number of participants with year-3 follow-up FAST scores available, leading to a subsample of 59 participants (41 amyloid positive, 18 amyloid negative). Spearman’s correlation was used to evaluate the association of plasma markers with year-3 FAST score. Thirty-two individuals (progressors) progressed to more severe stages at the follow-up visit (follow-up FAST > 3), and twenty-seven individuals (non-progressors) had the same or lower FAST scores at their year-3 follow-up visits (follow-up FAST ≤ 3). A group comparison of blood-based markers between progressors and non-progressors was conducted with the 2-sample *t*-test.

Considering that amyloid pathology is the defining signature of AD pathology and it is vital for early diagnosis of AD, it is important to test the ability of these markers in differentiating brain amyloid status (amyloid positive versus amyloid negative). A stepwise logistic regression analysis was conducted to determine the optimal set of peripheral markers in predicting brain amyloid status, starting from a model with only a constant (intercept) term and adding predictors if the *p* value of the chi-squared statistic is less than 0.05. The classification analysis was conducted among all participants or limited to cognitively impaired participants. Adjusting for covariates is not a common practice in machine learning; the classification analysis was carried out with the raw concentrations of plasma markers. The area under the receiver operating characteristic curve (AUC) was computed and used to compare the prediction performance. Bootstrapping with 1000 iterations was used to obtain the 95% confidence interval of the AUC values. The software Matlab R2022a (The MathWorks, Inc., Natick, MA, USA) was used to perform statistical analysis in the study.

## Results

In the linear regression model, plasma GFAP concentration was found to be significantly associated with amyloid positivity (*t* = 5.81, Bonferroni-corrected *p* < 0.001), age (*t* = 3.85, *p* < 0.001), and sex (*t* = 2.80, *p* = 0.02). Plasma ptau-181 was significantly associated with amyloid positivity (*t* = 5.42, *p* < 0.001) and sex (*t* =  − 2.50, *p* = 0.04). Plasma NfL was significantly associated with age (*t* = 6.62, *p* < 0.001). Clinical diagnosis did not show a significant main effect in the model; instead, the interaction term between clinical diagnosis and amyloid positivity was significant for plasma GFAP (*t* = 3.14, *p* = 0.006) and ptau-181 (*t* = 2.67, *p* = 0.03) but not for NfL. Education was not associated with any plasma markers.

### Group differences of plasma protein concentrations in participants stratified by clinical diagnosis and amyloid status

**Fig. 1:**
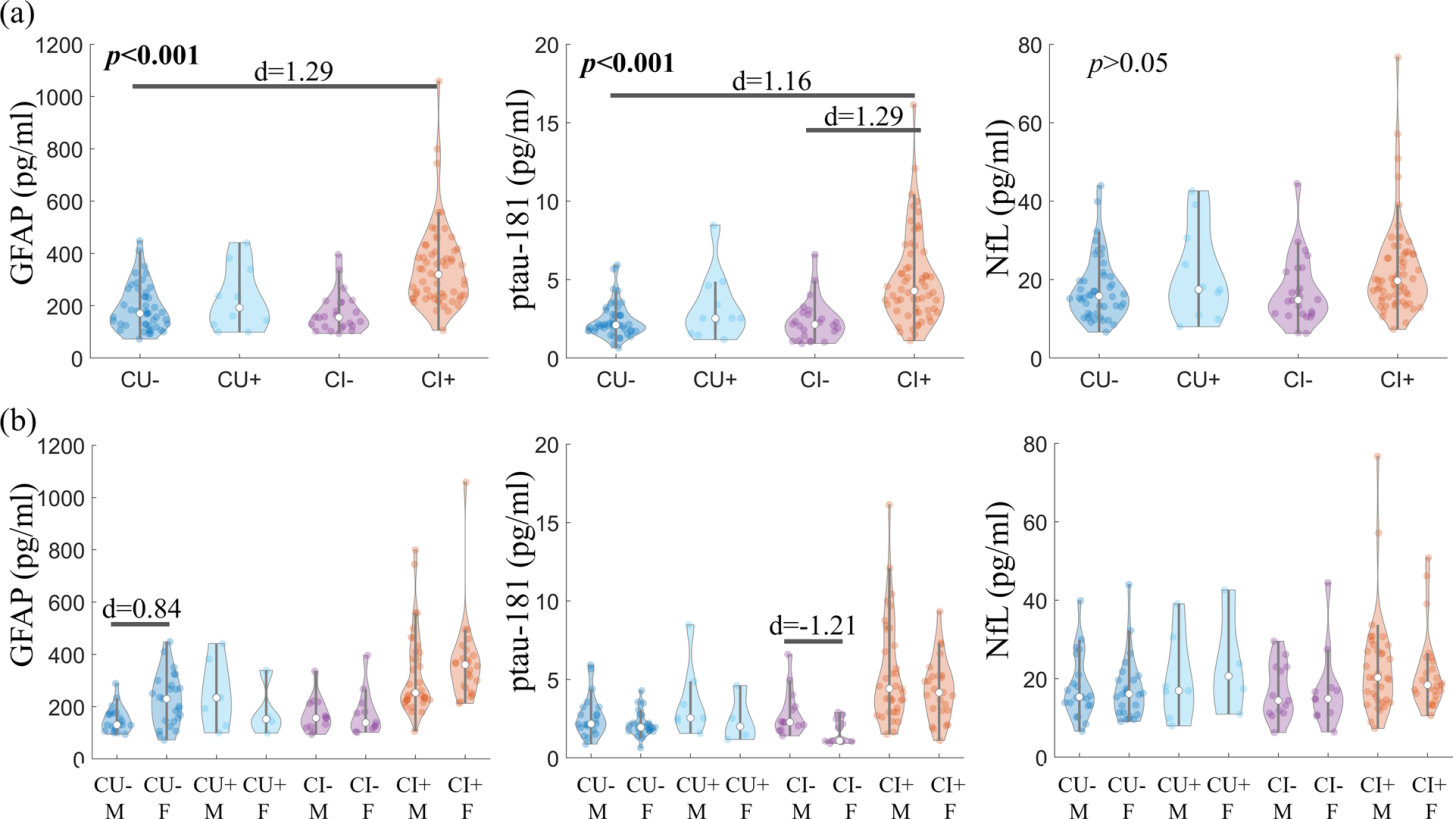
Group comparisons of GFAP, ptau-181, and NfL. **a** Violin plots of blood-based markers between groups stratified by clinical diagnosis and brain amyloid status. The comparisons between groups were conducted after adjusting for demographical variables including age, sex, and education. **b** Sex differences in each group. The comparisons between male and female were conducted separately for each group, and the concentrations were not adjusting for demographical variables since sex was the factor of interest. CU, cognitively unimpaired; CI, cognitively impaired; ± , brain amyloid positive/negative; M, male; F, female

The violin plots of GFAP, ptau-181, and NfL concentrations in the groups stratified by clinical diagnosis and amyloid status are shown in Fig. 1. The concentrations of plasma GFAP were 191.7 ± 88.1, 223.5 ± 117.2, 172.5 ± 75.3, and 347.5 ± 160.8 pg/ml for CU − , CU + , CI − , and CI + groups, respectively. In the post hoc analysis, CI + participants had significantly higher plasma GFAP and ptau-181 levels than both CI − and CU − participants with very large effect (Cohen’s *d* > 1.0, corrected *p* < 0.001). Compared to CU + group, the CI + group had higher GFAP with a large effect (Cohen’s *d* = 0.95) and higher ptau-181 with a moderate effect (Cohen’s *d* = 0.78), but the difference did not pass the significance level, possibly due to the small sample size of CU + group. Plasma NfL did not show differences in the pairwise group comparisons.

To further evaluate the role of sex on plasma markers, the differences of plasma GFAP, ptau-181, and NfL between female and male were tested separately in CU − , CU + , CI − , and CI + groups by 2-sample *t*-tests. In the CU − group (21 M/25F), female had significantly higher plasma GFAP level than male (Cohen’s *d* = 0.84, *p* = 0.02). In the CI − group (15 M/11F), male had significantly higher plasma ptau-181 level than female (Cohen’s *d* =  − 1.21, *p* = 0.03). All the other comparisons did not show significant differences between female and male.

**Fig. 2:**
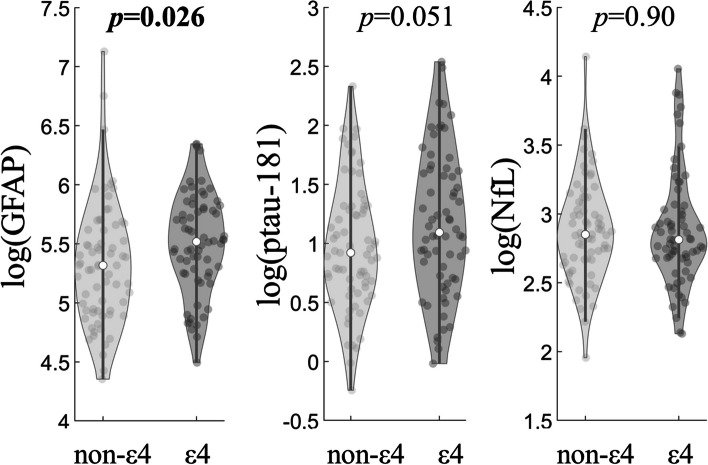
Group comparisons of blood-based markers between APOE ε4 carriers (39 M/27F) and non-ε4 carriers (34 M/35F) across all participants after adjusting for age, sex, and education. When the analysis was carried out with stratified amyloid status or clinical diagnosis, the difference was no longer observed for all three markers

The comparisons of plasma markers between ε4 carriers and non-ε4 carriers were shown in Fig. 2. Across all participants, the plasma GFAP was observed to be higher in the ε4 carriers (39 M/27F) than non-ε4 carriers (34 M/35F) with a small effect (Cohen’s *d* = 0.39, *p* = 0.026). Plasma ptau-181 (Cohen’s *d* = 0.34, *p* = 0.051) showed the trend towards significance level. The NfL concentration did not differ between non-ε4 and ε4 carriers (Cohen’s *d* = 0.02, *p* > 0.05). When the analysis was carried out with stratified amyloid status or clinical diagnosis, plasma GFAP no longer showed the difference between non-ε4 and ε4 carriers.

### Age effect on blood-based markers in normal aging and those with AD pathology

**Fig. 3:**
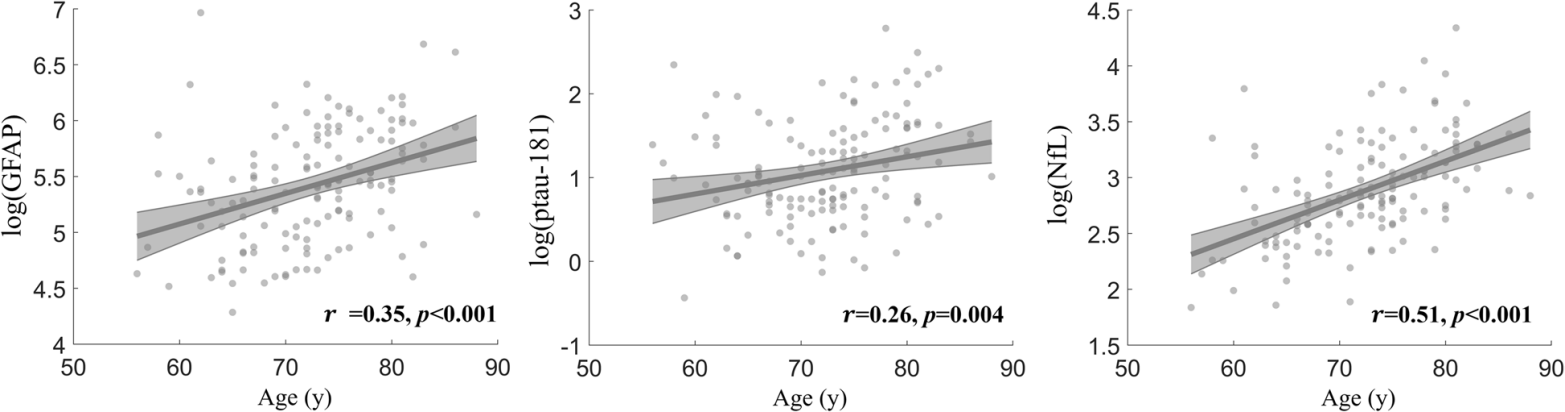
Linear fitting between age and blood-based biomarkers across all participants. Significant associations were still observed when blood-based biomarkers were adjusted for CDR score or AV45 composite SUVR. The analysis was carried out without adjusting for demographical variables since age was the factor of interest. Age explained 12%, 5%, and 26% of variance for plasma GFAP, ptau-181, and NfL respectively based on *r*^*2*^ value. The age effects on all three markers were not significantly different between CU − and Amy + (both CU + and CI + included) participants. The shaded area represents the 95% confidence interval of the fitting curve

Correlation analysis was applied separately for CU − and Amy + (both CU + and CI + included) participants to evaluate age effect on blood-based markers and determine if age effect is altered by AD pathology. Plasma NfL and GFAP were significantly correlated with age among CU − participants and the correlations became weaker or not significant among Amy + participants (see Supplementary Table 1); however, Fisher’s *z* statistic did not find significant correlation differences between these two groups (*p* > 0.05). Plasma ptau-181 did not show significant associations with age either within CU − or within Amy + participants. When the analysis was conducted across all participants, age was significantly associated with all three markers (see Fig. 3). NfL had 26% of variance (namely *r*^*2*^ value) explained by age (*r* = 0.51, *p* < 0.001), followed by GFAP (*r* = 0.35, *p* < 0.001) and ptau-181 (*r* = 0.26, *p* = 0.004). The associations remained consistent when the blood-based markers were adjusted for CDR score or AV45 composite SUVR.

### Blood-based markers in ATN framework

**Fig. 4:**
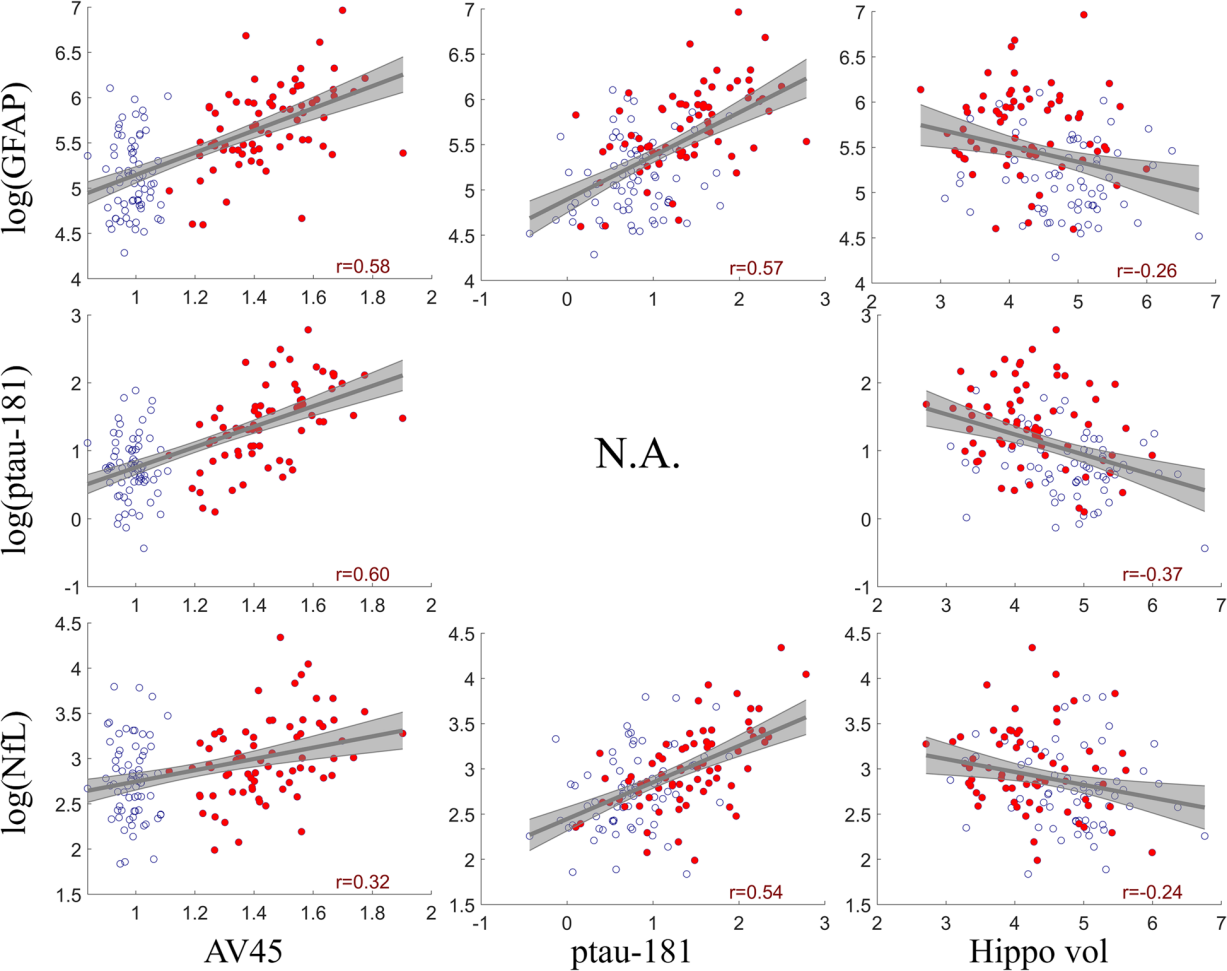
Correlation analysis of blood-based markers in the ATN framework for GFAP, ptau-181, and NfL from top to bottom. AV45 composite SUVR, ptau-181, and hippocampal volume were used as the A, T, and N marker, respectively. The red dots indicate amyloid positive participants and blue circles indicate amyloid negative participants. The linear fitting curves with 95% confidence interval were also shown in the figure. Since ptau-181 itself was treated as the T marker, the correlation between ptau-181 and T marker was meaningless and thus marked as not applicable (N.A.) in the figure. Spearman’s correlation was used for AV45 due to its non-normality and Pearson’s correlation was used for all other measures

The scatter plots between plasma proteins and ATN markers, namely AV45 composite SUVR, ptau-181, and hippocampal volume were shown in Fig. 4, with the correlation values marked in the figure. GFAP (first row) was strongly correlated with AV45 composite SUVR (*r* = 0.58, *p* < 0.001) and ptau-181 (*r* = 0.57, *p* < 0.001), followed by hippocampal volume (*r* =  − 0.26, *p* = 0.03). Plasma ptau-181 was significantly correlated to both AV45 composite SUVR (*r* = 0.60, *p* < 0.001) and hippocampal volume (*r* =  − 0.37, *p* < 0.001). NfL was the most strongly correlated to ptau-181 (*r* = 0.54, *p* < 0.001), followed by AV45 composite SUVR (*r* = 0.32, *p* < 0.001). The correlation between NfL and hippocampal volume was -0.24, which did not pass the multiple correction (*p* > 0.05). Separate correlation analysis among Amy + participants and among CU − participants showed that the associations of plasma makers with AV45 composite SUVR and ptau-181 were driven by Amy + participants (see Supplementary Figure 1).

### Clinical relevance of blood-based markers

**Fig. 5:**
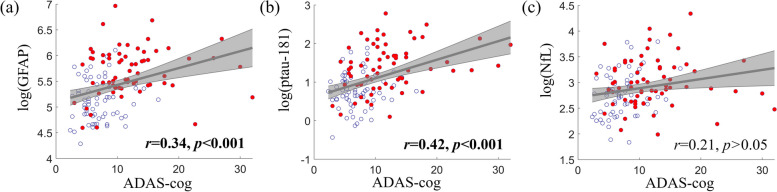
Clinical relevance of plasma ptau-181, NfL, and GFAP concentrations. The scatter plots of plasma GFAP (**(a)**), ptau-181 (**(b)**), and NfL (**(c)**) with ADAS-cog score across all participants were shown in the figure (blue circles: amyloid negative participants; red dots: amyloid positive participants). Bonferroni-corrected *p* values were marked in the figure

A higher ADAS-cog score (worse cognition) was associated with higher plasma ptau-181 (*r* = 0.42, *p* < 0.001; Fig. 5) and GFAP concentrations (*r* = 0.34, *p* < 0.001). The correlation of NfL with ADAS-cog was 0.21, which did not pass the multiple correction (uncorrected *p* = 0.018). Similar findings were observed when ADAS-cog was replaced by CDR-sum of boxes scores or the total MoCA scores for cognitive assessment in the analysis (not shown in the figure).

**Fig. 6:**
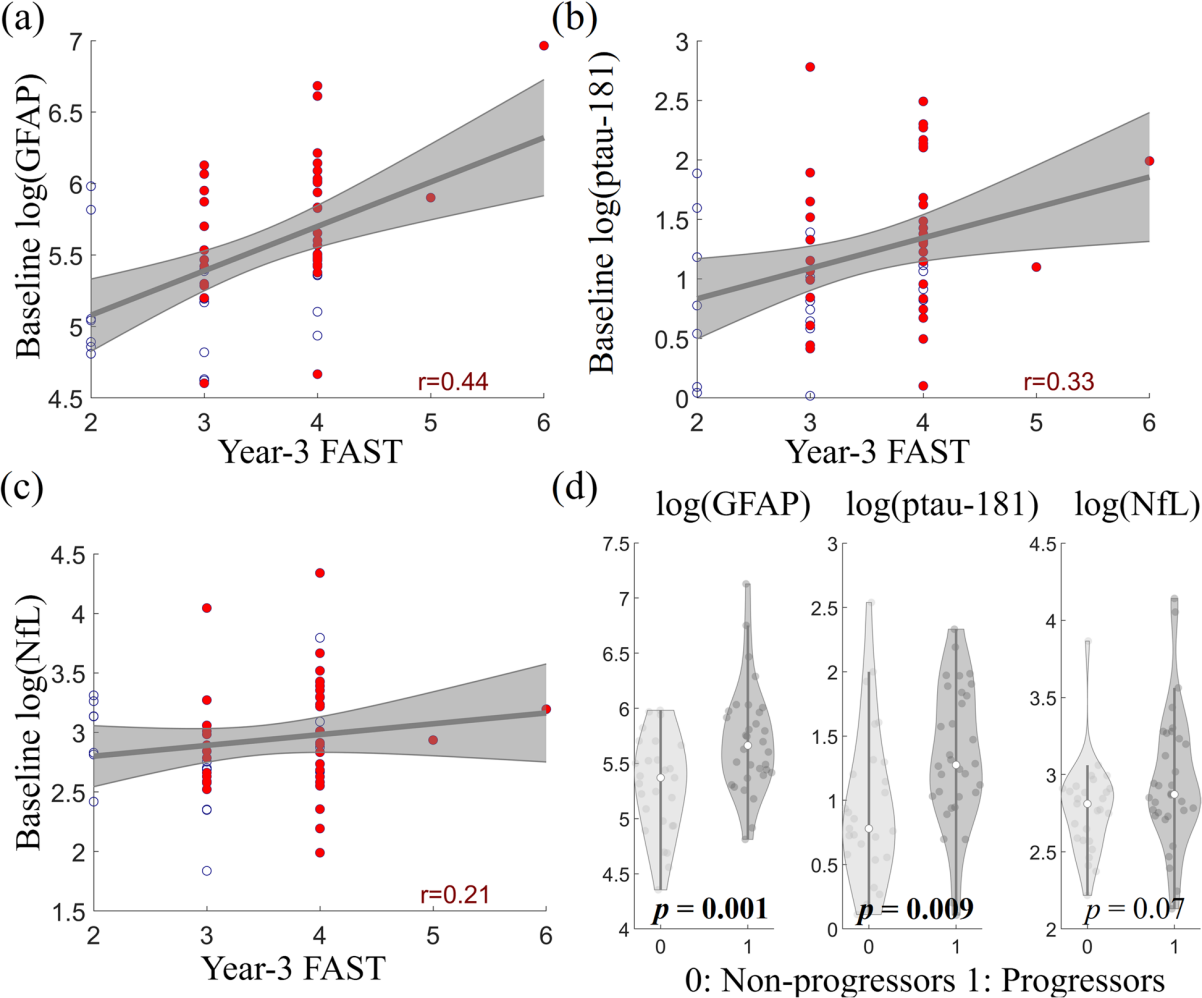
Prognostic power of plasma markers in predicting cognitive decline based on longitudinal FAST assessments. All participants included in the analysis had baseline FAST score as 3. Scatter plots between year-3 FAST score and plasma markers are shown for GFAP (**(a)**), ptau-181 (**(b)**), and NfL (**(c)**). Blue circles indicate amyloid negative participants and red dots indicate amyloid positive participants. **d** Comparisons of baseline plasma concentrations of GFAP, ptau-181, and NfL between progressors (year-3 FAST > 3) and non-progressors (year-3 FAST ≤ 3). The plasma marker levels were adjusted for age, sex, and education

To evaluate the prognostic power of these three plasma markers in predicting disease progression, we tested the relevance of baseline plasma markers with the year-3 FAST assessment among the 59 participants having the same baseline FAST score (baseline FAST = 3). Baseline plasma GFAP level was significantly correlated to year-3 FAST score (*r* = 0.44,* p* = 0.002, Fig. 6a); such a correlation remained consistent when the analysis was limited to amyloid positive individuals. Baseline plasma ptau-181 was correlated to year-3 FAST score with weaker strength (*r* = 0.33, *p* = 0.04, Fig. 6b), and the correlation was not significant within amyloid positive individuals. Baseline NfL was not correlated to year-3 FAST score (*r* = 0.21, *p* > 0.05, Fig. 6c). After removing the two outliers with FAST scores above 4, the correlation remained significant for GFAP (*r* = 0.40, *p* = 0.009) and was marginally significant for ptau-181 (*r* = 0.31, *p* = 0.06). When these participants were categorized into progressors and non-progressors based on the change of FAST score at year-3 visit, progressors (year-3 FAST > 3) had significantly higher GFAP (Cohen’s *d* = 0.88, *p* = 0.001, Fig. 6d) and ptau-181 (Cohen’s *d* = 0.70, *p* = 0.009) concentrations than non-progressors (year-3 FAST ≤ 3), and NfL showed (Cohen’s *d* = 0.48, *p* = 0.07) a trend towards significance.

### Blood-based markers to predict brain amyloid status

**Fig. 7:**
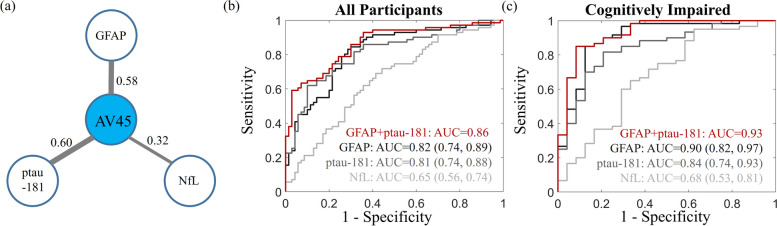
Classification of amyloid status by blood-based markers. **a** Correlation between AV45 composite SUVR and blood-based markers. Stepwise logistic regression showed that combining GFAP and ptau-181 together is the optimal model to predict brain amyloid status (amyloid positive versus amyloid negative). The receiver operating characteristic curves were shown for GFAP, ptau-181, and NfL separately or GFAP + ptau-181 (red). The analysis was carried out across all participants (**(b)**) or among cognitively impaired participants (**(c)**). The area under the curve (AUC) values and 95% confidence intervals were marked in the figure. Adding NfL as an additional predictor to the optimal model has a marginal improvement of AUC less than 0.01

The pairwise correlations between plasma fluid biomarkers and AV45 composite SUVR are shown in Fig. 7a with the linewidth indicating the correlation strength. The strong correlations, particularly the values with GFAP and ptau-181, suggest their potential utility in identifying subjects with abnormal brain amyloid. A logistic regression model was used to examine the capability of these markers in classifying brain amyloid status. When separate analyses were carried out for each marker across all participants, the AUC values for GFAP, ptau-181, and NfL were 0.82, 0.81, and 0.65, respectively (Fig. 7b). Improved performance was observed, particularly for GFAP, when the analysis was limited to cognitively impaired participants with AUC values as 0.90, 0.84, and 0.68 for GFAP, ptau-181, and NfL, respectively (Fig. 7c). The stepwise logistic regression model identified that combining GFAP and ptau-181 was the best model to distinguishing brain amyloid status, no matter the model was applied among all participants (AUC = 0.86) or within cognitively impaired participants (AUC = 0.93). Adding NfL as an additional predictor had a marginal effect with the AUC value improved by less than 0.01.

**Fig. 8:**
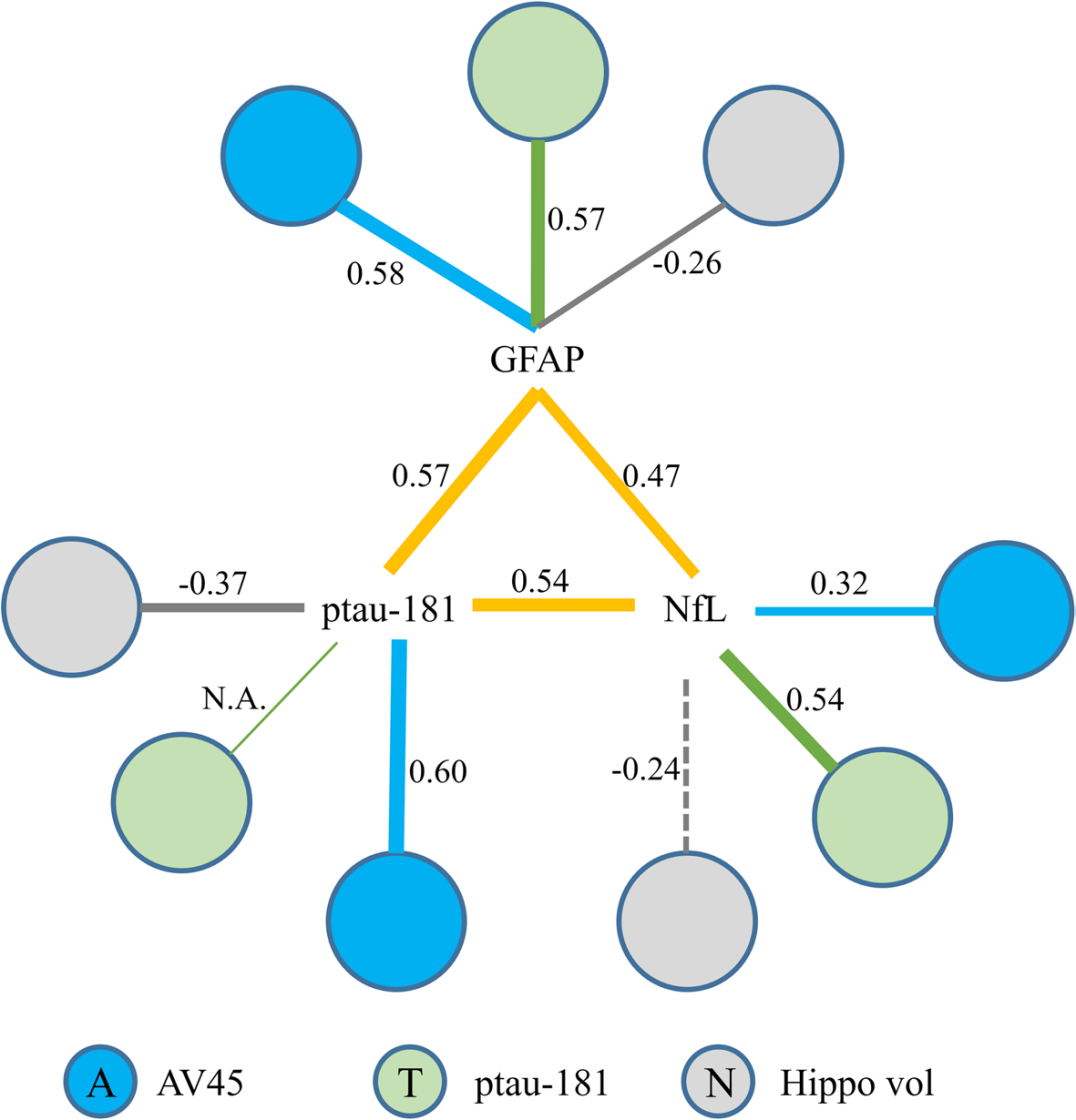
An overview of the correlations between blood-based markers (orange color) or between blood-based markers and ATN markers characterized by AV45 composite SUVR (blue color), ptau-181 (green color), and hippocampal volume (gray color), respectively. The correlation values were marked in the figure. Solid lines indicate significant correlations after Bonferroni correction. Dashed lines indicate insignificant correlations. Since ptau-181 itself was treated as the T marker, the correlation between ptau-181 and T marker was meaningless and thus marked as not applicable (N.A.) in the figure

Finally, the Pearson’s correlations among blood-based markers (orange color) or between blood-based markers and ATN markers, namely AV45 composite SUVR (blue color), ptau-181 (green color), and hippocampal volume (gray color), were summarized in Fig. 8. Significant correlations were marked with solid lines and insignificant correlations were marked with dashed lines. The linewidth is proportional to the correlation strength. Moderate correlations between GFAP, ptau-181, and NfL were observed.

## Discussion

There is an emerging demand for blood-based biomarkers that can be used reliably to screen for and eventually diagnose AD. The inclusion of blood-based biomarkers in clinical trials is vital for treatment monitoring and the evaluation of novel therapeutics. The data in the study were collected at a single site with a moderate sample size, which could contribute as an independent set for AD research. A comprehensive collection of neuropsychological tests, blood samples, and MRI/PET scans in the cohort add to the growing interest of investigating the relevance of blood-based biomarkers with AD clinical and/or imaging measures [24, 25]. The strong association of plasma GFAP with brain amyloid pathology suggests its potential utility for screening amyloid pathology, with properly adjusting for age effect recommended. Its small-to-moderate correlations with hippocampal volume and cognitive scores indicates that plasma GFAP is not a robust indicator of neurodegeneration and cognitive impairment; however, plasma GFAP might be of prognostic value in predicting future cognitive decline.

Compared to plasma NfL, both GFAP and ptau-181 had much stronger statistical power in differing the groups stratified by clinical diagnosis and amyloid positivity. More specifically, GFAP and ptau-181 were substantially elevated in CI + participants compared to CU − and CI − participants. Similar group differences were observed in other studies [15, 26], demonstrating the potential utility of both markers. NfL showed similar trend as GFAP and ptau-181, but the group differences did not reach equivalent strength. It may be because the majority of participants in CI + are prodromal AD (namely MCI) instead of AD dementia; thus NfL, as a marker of neurodegeneration manifested at the late stage of the disease, was not sufficiently elevated yet in our cohort. APOE ε4 allele is the strongest genetic risk factor in sporadic AD [27, 28]. Plasma GFAP level differed between ε4 and non-ε4 carriers across all participants with weak-to-moderate effect, but such a difference no longer existed when the analysis was conducted with stratified amyloid positivity or clinical diagnosis. This finding suggested that the presence of ε4 allele does not have additive effect on influencing plasma GFAP concentration beyond amyloid load, which is expected since APOE ε4 and amyloid load are the confounding factors of each other possibly due to their close interaction [29].

Several previous reports have demonstrated age associated with changes in plasma NfL and GFAP [30, 31], although age effect on NfL concentration does not predict cognitive decline or AD [32]. Similar to previous report [26, 33, 34], elevated plasma NfL concentration was observed to be strongly associated with older age in the study, followed by GFAP and ptau-181 at much weaker extent. In addition, although the associations with age were relatively weaker for the participants under AD pathology compared to CU − group, such a difference did not reach the significance level in our study. A previous study showed that plasma GFAP, ptau-181 and NfL had weaker associations in early and late onset sporadic AD cases compared to elderly controls [33]. Another study found that amyloid positive MCI group had stronger correlation between plasma GFAP and age than amyloid negative MCI group [35]. But whether the differences reached significance level were not tested in these studies. The influence of AD pathology on blood-based markers might dilate the contribution made by age, which could be the reason leading to the findings in these studies. Collectively, regardless if an individual is under AD pathology, chronologic age remains to be a factor influencing the concentrations of plasma markers. These plasma markers could be less discriminative between AD disease cases and controls when the disease cases were younger than the controls. Properly adjusting for age would be required when using these markers, plasma NfL in particular, for screening or other clinical utilities.

In the groups stratified by clinical diagnosis and amyloid status, plasma GFAP level was higher in female than male only in the CU − group, and similar trend was observed in the CI + group but did not reach significance level. Higher plasma GFAP in female than male was suggested in previous studies with various populations [36, 37]. Larger number of CI + individuals could help to test if plasma GFAP remains differed by sex for the ones under AD pathology. Plasma ptau-181 was higher in male than female only in CI − group, but not in any other groups. A previous study also showed that there was no difference of plasma ptau-181 between female and male in CI + group [38]. The CI − individuals are not on the AD continuum in the NIA-AA research framework, such a difference might be caused by non-AD related pathology.

The data from the present study highlight a promising role of GFAP as a candidate biomarker for screening and eventual diagnosis, in isolation or combination with other markers. The association analysis of GFAP within the NIA-AA ATN research framework was conducted to illustrate the relevance of GFAP in AD from the biological aspect. GFAP was most strongly correlated to amyloid-PET (A), followed by plasma ptau-181 (T and potentially A) and hippocampal volume (a marker of N). The substantial correlation between plasma GFAP and brain amyloid PET data was consistently found with various other ligands besides 18F-AV45, including 11C-PiB [16, 39–41], 18F-flutemetamol [15, 16], and 18F-florbetaben [16]. In fact, plasma GFAP was illustrated to outperform CSF GFAP in assessing amyloid pathology with three different cohorts [26]. The moderate association of plasma GFAP with plasma ptau-181 observed in the study was in concordance with previously reported association with tau-PET and CSF ptau-181 levels [15, 35, 36, 42], and such an association might be substantially mediated by amyloid pathology [36]. The statistical power of plasma GFAP in predicting amyloid positivity largely varied at different cohorts with AUC varying from 0.69 to 0.97 [15, 26, 36, 43, 44]; our study supported the robustness of plasma GFAP in predicting brain amyloid with an independent cohort. What is particularly encouraging is that the combination of GFAP and ptau-181 was strongly predictive of brain amyloid status. GFAP and ptau-181 were the primary driving factors to distinguish amyloid positive participants from amyloid negative participants. Consistent with previous studies, including NfL as an additional predictor resulted in a marginal improvement of the prediction [30, 36, 43]. The result suggests that, instead of using a single plasma marker alone, a combination of plasma markers GFAP and ptau-181 better predicts amyloid positivity for screening a large-scale population. Whereas GFAP and ptau-181 showed comparable power when the prediction was conducted in the entire cohort, GFAP showed superior performance compared ptau-181 when the participants were limited to cognitively impaired individuals. Plasma GFAP, ptau-181, and NfL were only moderately correlated to each other; they have demonstrated discrepant associations with brain amyloid (GFAP and ptau-181 versus NfL) and hippocampal volume (ptau-181 versus GFAP and NfL). In addition, the concentrations of these plasma markers were associated with demographical variables including age and sex in distinct ways. These data collectively support the relevant but differential values of plasma markers in AD assessment and the utility of multiple markers in a combinatorial approach for screening and eventual diagnosis of AD.

From the clinical aspect, the result suggested the potential value of blood-based biomarkers in clinical assessments and predicting future cognitive decline. All three blood-based markers showed small-to-moderate correlations with ADAS-cog. Among the participants having the same baseline FAST score, plasma GFAP level at the baseline visit was demonstrated to have the most striking association with the year-3 FAST score compared to ptau-181 and NfL across all these participants or within amyloid positive individuals, suggesting its valuable prognostic power in predicting cognitive decline. Plasma GFAP and ptau-181 concentrations at the baseline visit were observed to have a moderate-to-large effect in distinguishing the participants who progressed to worse functional status at year-3 follow-up from the ones who remained stable.

There are a few limitations with the study. First, despite moderate sample size of the entire cohort, the number of participants in CU + group is very limited, which makes it challenging to have enough statistical power to assess the sex difference in the preclinical AD population. Because of similar reason, only the participants with baseline FAST as 3 were used to assess the prognostic power of plasma markers. It remained to be investigated if the findings could be generalized to individuals at different functional status. Second, tau-PET imaging data were not available in the study, and the plasma ptau-181 concentration was treated as a marker of tau pathology in the association analysis with the ATN framework. Previous investigations have shown that one fifth of participants had discordant tau status determined by plasma ptau-181 and tau-PET scans [45]. An association analysis between GFAP and tau-PET would be required to consolidate our finding. Third, although plasma GFAP was consistently observed to elevate in participants with AD pathology, its concentration could substantially differ between studies for the same disease populations [34, 36, 46], which is possibly due to pre-analytical sample handling effects. The large inter-study variability is not unique to GFAP but is a general issue for other plasma markers, including ptau-181 and NfL, which makes the plasma marker levels not comparable between studies and could hinder the implementation of gold standards of plasma markers for their clinical applications.

In summary, our result supported that elevated plasma GFAP might be a collective effect of the complete ATN pathology. GFAP and ptau-181 played substantial and complementary roles in predicting brain amyloid status, with the advantage of GFAP over ptau-181 present in the cognitive impaired population. In addition, age, and possibly sex, was observed to influence the concentrations of these plasma markers in various degrees independent of AD pathology, suggesting that properly adjusting for demographical factors might be required when using these plasma markers for screening or clinical assessment.

### Supplementary Information

**Additional file 1: Supplementary Figure 1.** Correlation analysis.

**Additional file 2: Supplementary Table 1.** Linear regression model between age and plasma markers with the formula plasma marker ~ 1 + β*age separately for CU− and Amy+ participants.

## Data Availability

The dataset supporting the conclusions of this article could be requested through Nevada CNTN website (https://nevadacntn.org/).
