# Supplementary figures and images for: Clinical and biological relevance of glial fibrillary acidic protein in Alzheimer’s disease
Source: Alzheimers Res Ther. 2023 Nov 3;15:190. doi: 10.1186/s13195-023-01340-4 (PMC10623866; doi:10.1186/s13195-023-01340-4)

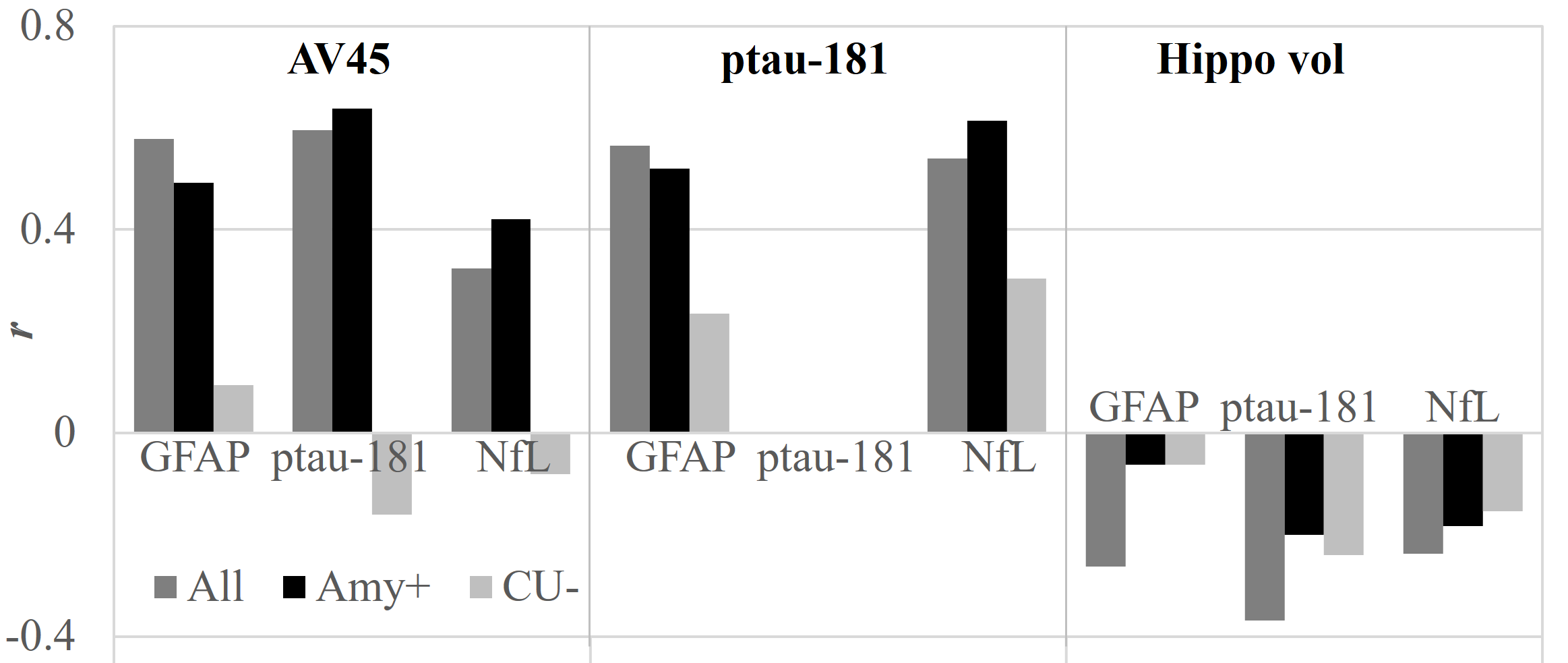

Supplement: Supplementary file 1 — Additional file 1: Supplementary Figure 1. Correlation analysis. [file 13195_2023_1340_MOESM1_ESM.tif]
