# Supplementary material for: Clinical and biological relevance of glial fibrillary acidic protein in Alzheimer’s disease
Source: Alzheimers Res Ther. 2023 Nov 3;15:190. doi: 10.1186/s13195-023-01340-4 (PMC10623866; doi:10.1186/s13195-023-01340-4)
Supplement: Supplementary file 2 — Additional file 2: Supplementary Table 1. Linear regression model between age and plasma markers with the formula plasma marker ~ 1 + β*age separately for CU− and Amy+ participants. [file 13195_2023_1340_MOESM2_ESM.docx]

Supp. Table 1: Linear regression model between age and plasma markers with the formula plasma marker ~ 1 + β*age separately for CU- and Amy+ participants.

|  | CU- | | | Amy+ | | | All | | |
| --- | --- | --- | --- | --- | --- | --- | --- | --- | --- |
|  | β | t value | p value | β | t value | p value | β | t value | p value |
| GFAP | 0.023 | 2.47 | 0.017 | 0.014 | 1.74 | 0.09 | 0.027 | 4.46 | <0.001 |
| ptau-181 | 0.011 | 1.11 | 0.27 | 0.012 | 1.16 | 0.25 | 0.022 | 2.97 | 0.0035 |
| NfL | 0.036 | 4.56 | <0.001 | 0.029 | 3.83 | <0.001 | 0.035 | 7.02 | <0.001 |
